# Superficial temporal artery aneurysm misdiagnosed in a patient with long-term migraine headache treatment: a case report

**DOI:** 10.1186/s13256-024-04647-4

**Published:** 2024-07-26

**Authors:** Meghdad Ghasemi Gorji, Ali Rafiei, Amirhossein Rajabi

**Affiliations:** 1 Department of Vascular Surgery, Shiraz University of Medical Science, Shiraz, Iran; 2 Student Research Committee, Shiraz University of Medical Sciences, Shiraz, Iran

**Keywords:** Superficial temporal artery aneurysm, Vascular surgery, Headache etiology, Case report

## Abstract

**Background:**

Superficial temporal artery aneurysm is a rare vascular abnormality without specific clinical symptoms. In this case report, we present the case of a patient with superficial temporal artery aneurysm who was diagnosed with migraine headache at first.

**Case presentation:**

A 60-year-old Iranian man with a previous history of headaches, who did not respond properly to the treatments following the initial diagnosis of migraine, presented with a painless lump in the left temporal region, and he was diagnosed with superficial temporal artery aneurysm via Doppler ultrasound. Finally, surgical removal of the left superficial temporal artery aneurysm was performed.

**Conclusions:**

This case shows the importance of vascular causes in the approach to headache etiologies, especially when the headache is prolonged without proper responses to treatment. Computed tomography angiography and magnetic resonance angiography are appropriate diagnostic methods for aneurysm detection that should be considered in future studies.

## Introduction

Superficial temporal artery (STA) aneurysm is a highly uncommon vascular abnormality that can be divided into true and false aneurysms. The majority of cases are classified as pseudoaneurysms and are often associated with blunt trauma. However, the spontaneous development of true STA aneurysms is a rare phenomenon, and the underlying mechanism leading to their development remains largely unknown [1]. Headache is one of the rarest symptoms associated with true STA aneurysm. However, accurate diagnosis of headache etiologies is sometimes challenging, resulting in inappropriate use of diagnostic resources, increased incidence of illness and mortality, and higher costs in establishing the correct diagnosis [2, 3]. In this case report, we present a patient who was diagnosed with migraine headache despite having a true STA aneurysm.

## Case presentation

**Fig. 1:**
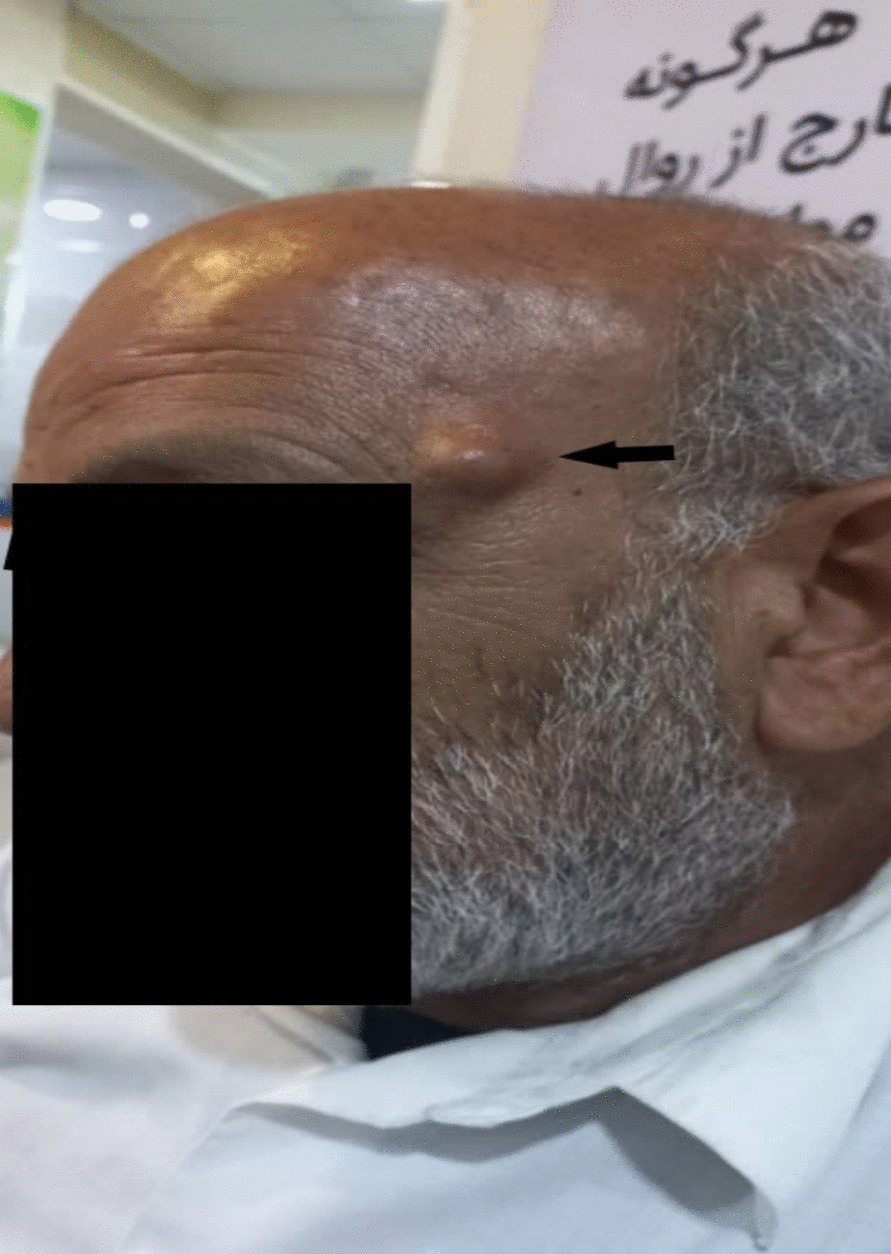
A painless lump has been noticed on the left temporal region

**Fig. 2:**
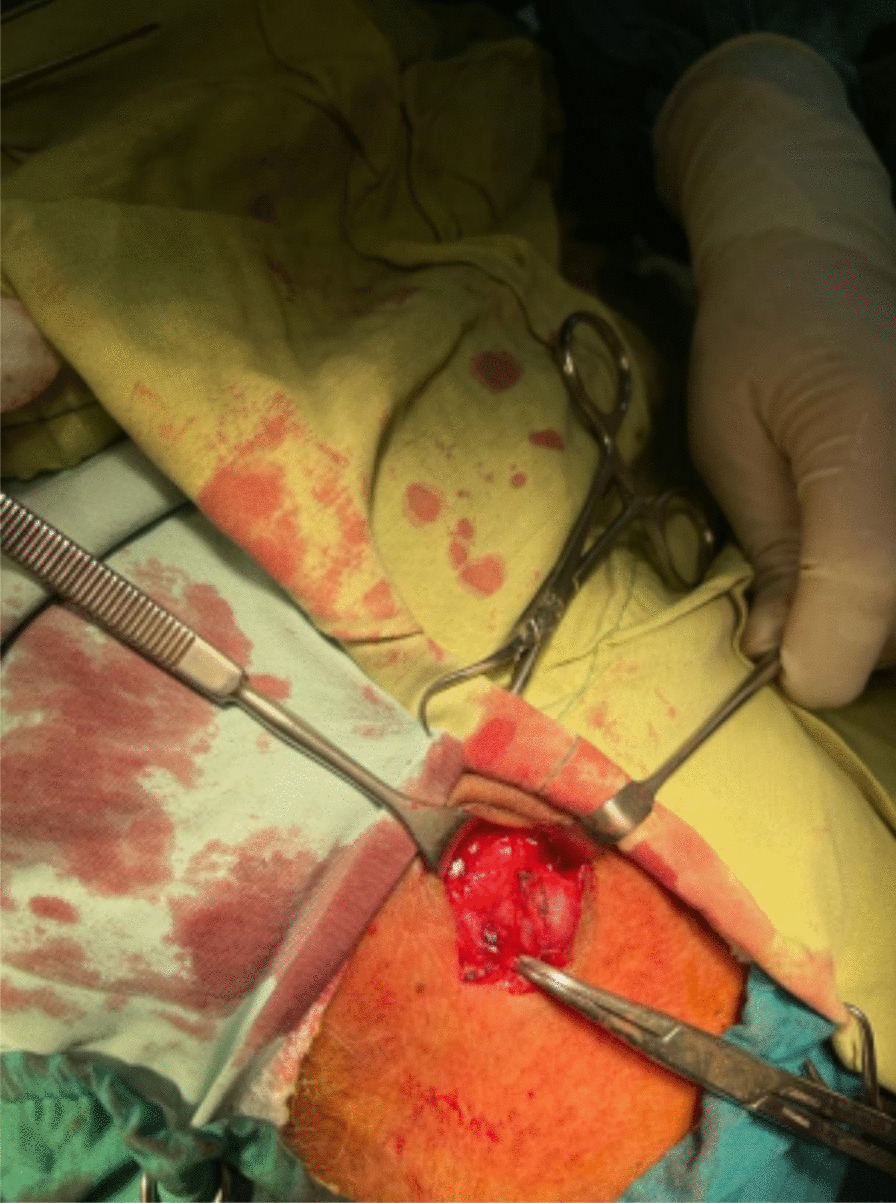
The superficial temporal artery aneurysm was clearly visible during the surgery

A 60-year-old Iranian male patient was referred to the Motahari clinic (Shiraz, Iran) by his family physician owing to a painless lump that had grown in his left temporal region (Fig. 1). The patient initially noticed the mass approximately 7 years ago but chose to dismiss it due to its small size and painlessness. Nevertheless, the lump exhibited gradual progression in size over the years. He denies any history of trauma to his head or face. Additionally, the patient reported experiencing intermittent headaches for about 5 years. He has been receiving medical treatment for a prolonged time for recurring headaches diagnosed as migraine. Unfortunately, the patient exhibited an inadequate response to the treatment administered. Upon examination, a nontender, pulsatile mass with a diameter of 1 cm was palpated that did not subside upon pressure on the proximal segment of the superficial temporal artery. An ultrasound examination of the soft tissue revealed a round lesion measuring 11.5 × 8.5 mm in size that appeared to be connected to the frontal branch of the left superficial temporal artery. Additionally, a color Doppler study demonstrated vascularity, leading to the diagnosis of a superficial temporal artery aneurysm. Surgical removal of the left STA aneurysm was performed (Fig. 2). The pathology report for the resected STA aneurysm indicated that the intima, media, and adventitia were well preserved. The patient did not report any headaches after the operation. On follow-up a month later, no complaints were mentioned.

## Discussion

An aneurysm is the abnormal dilation of a particular blood vessel in a localized area. The reason for most aneurysms observed in large- to medium-sized arteries is unclear, as only a small fraction have identifiable pathological causes. Inflammation, upregulation of proteolytic pathways, and loss of arterial wall matrix are some of the pathological mechanisms that contribute to the development of most degenerative aneurysms. The prevalence of aneurysms varies depending on the location. For instance, aneurysms are frequently observed in some body regions, including the abdominal aorta, but infrequently in other areas, such as the external iliac artery. According to the literature, the superficial temporal artery is one of the rarest locations for arterial aneurysms, which are often post-traumatic pseudoaneurysms.

A true STA aneurysm is even rarer than an STA pseudoaneurysm. After reviewing relevant literature, it can be concluded that an STA aneurysm alone is not usually life-threatening. However, it is essential to recognize the potential clinical significance of having aneurysms in multiple anatomical regions. According to a study by Norman *et al.* in 2010, autopsies found that nearly 40% of men and 25% of women with a thoracic aneurysm also had coexisting abdominal aortic, iliac, or femoral aneurysms. Additionally, around 7% of patients with abdominal aortic aneurysms (AAAs) and 5% with thoracic aortic aneurysms (TAAs) were found to have a cerebral aneurysm, suggesting a weak association between them [4]. The occurrence of simultaneous intracranial aneurysms (IA) in patients with true spontaneous aneurysm of STA was first reported by Ohta *et al.* in 2003 [5]. Subsequently, there have been reports of three more cases with concurrent aneurysms [6–8]. It is noteworthy that, in all four documented cases, two patients experienced subarachnoid hemorrhage (SAH) in connection with the coexistence of IA and STAA.

## Conclusions

Therefore, it is reasonable to investigate the presence of aneurysms in other body regions when STA aneurysms are detected take appropriate preventive measures and avoid potential complications. Computed tomography angiography (CTA) and magnetic resonance angiography (MRA) are appropriate diagnostic methods for detecting STA aneurysms, which is reasonable due to the possible coexisting aneurysms in different locations, such as cerebral arteries.

In our case, the patient complained of prolonged headaches that did not respond well to migraine treatment. Therefore, conducting a CTA and brain magnetic resonance imaging (MRI) was deemed appropriate to investigate other potential causes. Despite the tests being normal for intracranial lesions, they were essential to rule out any other conditions that could be causing the patient’s headache.

## Data Availability

Not applicable.

## Abbreviations

STA: Superficial temporal artery
STAA: Superficial temporal artery aneurysm
CTA: Computed tomography angiography
MRA: Magnetic resonance angiography
